# Viruses Utilize Cellular Cues in Distinct Combination to Undergo Systematic Priming and Uncoating

**DOI:** 10.1371/journal.ppat.1005467

**Published:** 2016-04-07

**Authors:** Madhu Sudhan Ravindran, Billy Tsai

**Affiliations:** Department of Cell and Developmental Biology, University of Michigan Medical School, Ann Arbor, Michigan, United States of America; University of Florida, UNITED STATES

**Fig 1:**
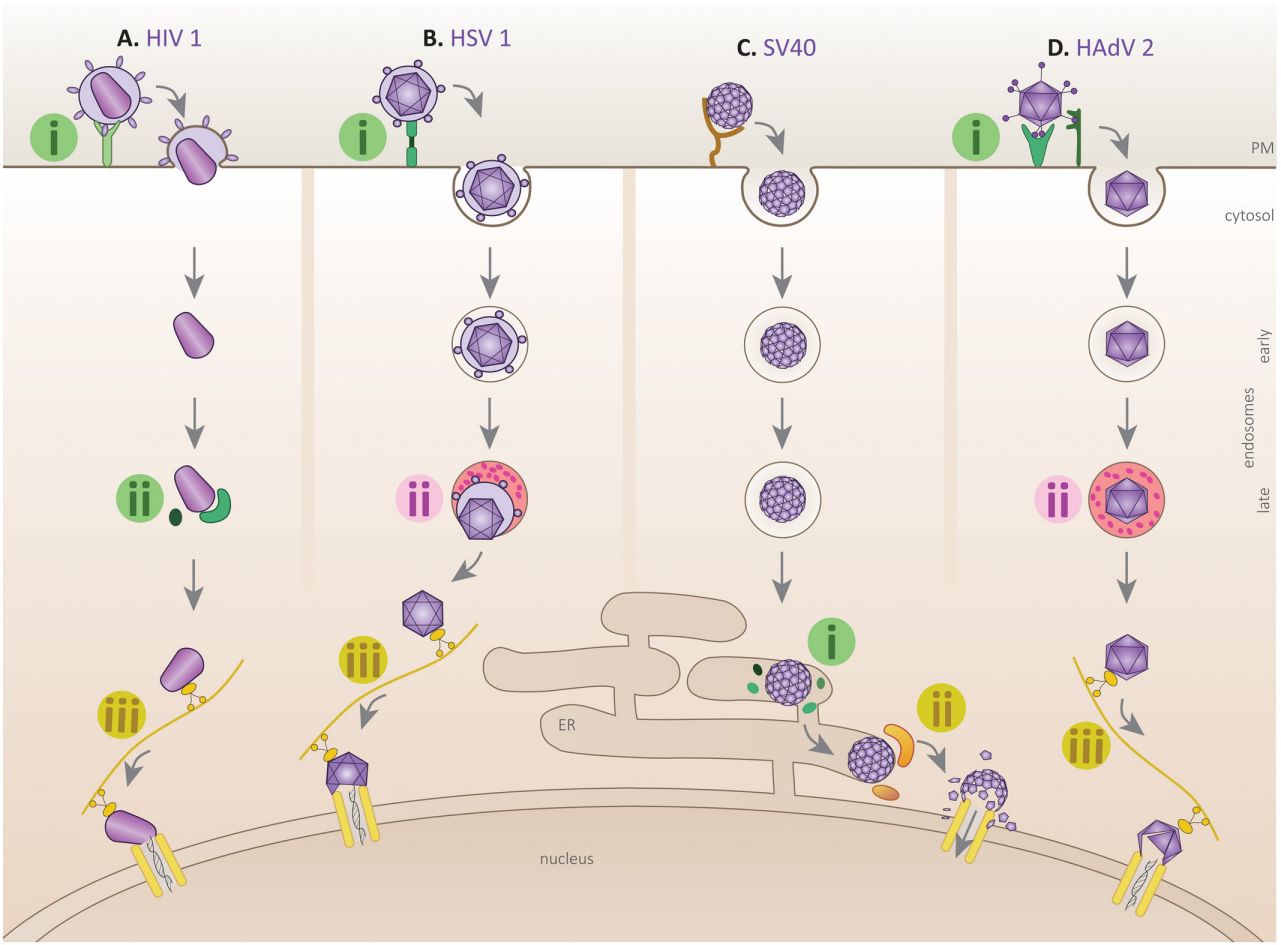
Virus utilizes host cues in distinct combination to uncoat. **(A)** Receptor–Enzyme–Mechanical: HIV-1 binding to its receptor structurally alters GP120, inducing membrane fusion (step i) and capsid release into the cytosol. Cytosolic peptidyl-isomerase conformationally alters the capsid (step ii), which is then trafficked to the nuclear pore by motor proteins to execute mechanical disassembly (step iii). **(B)** Receptor–Chemical–Mechanical: Herpes simplex virus-1 (HSV-1) engagement to its receptors alters the structural proteins (step i), which then induce endocytosis. The low pH endocytic compartment further alters the structural proteins (step ii) to promote fusion and capsid escape into the cytosol, where engagement with motor protein causes disassembly (step iii). **(C)** Enzyme–Mechanical: SV40 binds to its glycolipid receptor and reaches the endoplasmic reticulum (ER) unaltered via endocytic route. In the ER, the protein disulfide isomerase (PDI)-family of isomerases/reductases rearrange the disulphide bonds (step i) to structurally alter the virus. The viral capsid is then engaged by cytosolic disaggregation machinery (step ii), which extracts and simultaneously disassembles the viral particle. **(D)** Receptor–Chemical–Mechanical: Binding of human adenovirus-2 (HAdV2) to its receptors imposes mechanical strain due to drifting motion of the receptors (step i). The destabilized virus undergoes further structural distortion at low endosomal pH, which probably assists in capsid release into the cytosol (step ii). In the cytosol, the destabilized capsid engages the motor protein, which transports the capsid to the nuclear pore to undergo mechanical disruption (step iii), leading to genome release. Note: small Roman numerals (i, ii, and iii) represent virus coopting host cues. The background colors of the Roman numerals categorize them into receptor or enzyme (green), chemical (red), and mechanical (yellow).

Viral genomes are protected within a proteinaceous shell called “capsid” and, for enveloped viruses, an additional lipid coat. The capsids are generally constructed from a few capsid proteins into helical or icosahedral structures that are, in turn, stabilized by numerous covalent and noncovalent interactions [1]. However, during infection, viruses must uncoat in order to release their genomes into the host. This process is highly dependent on host elements called “cues” [2], which have been previously broadly categorized as (1) receptor- and/or enzyme-based cues, (2) chemical cues, or (3) mechanical cues [3]. In this brief article, we systematically analyze the available information on how 30 different enveloped and nonenveloped viruses exploit these host cues during infection and tabulate the observations in Table 1. By categorizing these cues, a general pattern can be deduced. Specifically, we find that these viruses use a distinct order and combination of the host cues during entry. To illustrate this principle, the mechanism by which four viruses hijack these cues will be highlighted (in Fig 1). We envision such analysis will provide an opportunity for investigators to evaluate whether viruses within the same family—for which the uncoating mechanism is unknown—employ a similar uncoating strategy.

**Table 1:** Virus uncoating and host cues.

family	strain	extracellular priming	intracellular priming and disassembly	
		receptor, enzyme	chemical or enzyme, chaperone	mechanical
**Enveloped virus**				
*Reteroviridae*	Human immunodeficiency virus 1	receptor	enzyme	motor
*Reteroviridae*	Avian leukosis virus	receptor	low pH	ND
*Herpesviridae*	Herpes simplex virus 1	receptor	low pH	motor
*Poxviridae*	Vaccinia virus	NA	low pH	proteasome
*Asfarviridae*	African swine flu virus	ND	low pH	motor
*Alphaviridae*	Semliki forest virus	NA	low pH	ribosome
*Orthomyxoviridae*	Influenza virus	receptor	low pH	NA
*Filoviridae*	Ebola virus	receptor	enzyme **	ND
*Hepadnaviridae*	Hepatitis B virus	protease	enzyme **	ND
*Coronaviridae*	Mouse hepatitis virus 2/4	protease, receptor	enzyme **	ND
*Coronaviridae*	SARS-coronavirus	protease, receptor	enzyme **	ND
*Paramyxoviridae*	Hendra virus	receptor	enzyme **	ND
*Paramyxoviridae*	Nipah virus	ND	enzyme **	ND
**Nonenveloped virus**				
*Papillomaviridae*	Human papillomavirus 16	receptor, enzyme	low pH	disaggregation machinery
*Parvoviridae*	Adeno-associated virus 2/8	NA	enzyme **	proteasome
*Parvoviridae*	Minute virus of mice	NA	low pH	proteasome
*Parvoviridae*	Canine parvovirus	NA	low pH	proteasome, motor
*Polyomaviridae*	Simian virus 40	NA	enzyme, chaperone	disaggregation machinery
*Polyomaviridae*	Mouse polyomavirus	NA	enzyme, chaperone	ND
*Polyomaviridae*	John Cunningham virus	NA	enzyme, chaperone	ND
*Polyomaviridae*	BK virus	NA	enzyme, chaperone	ND
*Adenoviridae*	Human adenovirus 2/5	receptor *	low pH	motor
*Picornaviridae*	Human rhinovirus 14/3	receptor	low pH	ND
*Picornaviridae*	Human rhinovirus 1/2/16	NA	low pH	ND
*Picornaviridae*	Poliovirus	receptor	ND	NA
*Picornaviridae*	Cosackie B3 virus	receptor	low pH	NA
*Picornaviridae*	Foot-mouth disease virus	NA	low pH	NA
*Picornaviridae*	Equine rhinitis A virus	NA	low pH	NA
*Reoviridae*	Reovirus 3	receptor	enzyme **	NA
*Reoviridae*	Rotavirus	protease, receptor	low pH, Ca^2+^	NA

*Receptor and/or enzyme-based cues*: receptor, enzyme, chaperone. *Chemical cues*: low pH, Ca^2+^. *Mechanical cues*: Motor, disaggregation machinery, proteasome, ribosome. NA: not applicable; ND: not determined.

* receptor- and coreceptor-induced mechanical stress;

** pH-dependent enzyme-induced priming.

One virus whose entry mechanism has been intensely studied is HIV-1, a *Retroviridae* enveloped RNA virus that causes the devastating acquired immune deficiency syndrome (AIDS). Viral entry commences when the viral envelope glycoprotein GP120 binds to the host surface glycoprotein receptor CD4 and coreceptor CCR5/CXCR4. This causes GP120 to undergo structural alterations that promote membrane fusion (Fig 1A, step i) [4]. After fusion, the HIV-1 capsid core is released into the cytosol, where reverse transcription of its RNA genome is initiated. This is followed by a two-stage viral disassembly process: a loss of core integrity followed by viral genome release. In the first stage, the cytosolic peptidyl-prolyl isomerase (cyclophilin A) catalyzes isomerization of peptide bonds between the capsid proteins, inducing a conformational change that causes capsid disintegration (step ii) [5]. Mechanical disruption by motor proteins (dynein and kinesin) near the nuclear pore ensues [6], thereby liberating the newly reverse-transcribed DNA into the nucleus (step iii). Thus, HIV-1 multistep uncoating requires the coordinated use of host receptor, enzyme, and mechanical cues leading to genome release.

Another well-characterized viral entry strategy is seen in the Herpes simplex virus-1 (HSV-1), a member of the *Herpesviridae* enveloped DNA virus family that can cause either lytic or latent infections. While HSV-1 entry shares similarities to HIV-1, there is also a clear difference. Upon interaction with the TNF superfamily receptor, HSV-1 envelope glycoprotein gD undergoes conformational changes to promote endocytosis (Fig 1B, step i) [7]. The low endosomal pH in turn triggers additional structural alterations to the viral glycoprotein gB, promoting fusion of viral and endosomal membranes that releases the capsid into the cytosol (step ii) [8]; HIV-1 entry, by contrast, is thought to be pH-independent [9]. For HSV-1, the action of molecular motors (dynein and kinesin) at the nuclear pore is essential to disassemble and release the viral genome (step iii) [10]. It should be noted that the entry mechanisms of HIV-1 and HSV-1 have been reported to be cell-type specific [11,12]. Nonetheless, unlike HIV’s use of receptor-enzyme-mechanical cues, HSV-1 uses a modified combination, in which receptor-chemical-mechanical cues are instead exploited to deliver the viral genome into the host.

Remarkably, receptor engagement at the plasma membrane does not appear to initiate uncoating of *Polyomaviridae*, a nonenveloped DNA virus responsible for many human diseases ranging from nephropathy to cancer. In fact, for members of this virus family, such as the archetype SV40, uncoating is initiated in the endoplasmic reticulum (ER). Specifically, upon endocytosis, SV40 is routed to the ER, where protein disulfide isomerase (PDI) members isomerize and reduce the viral capsid disulfide bonds (Fig 1C, step i). These reactions destabilize the capsid and expose the hidden hydrophobic proteins VP2/3, allowing the virus to insert into the ER membrane [13]. The membrane-inserted virus subsequently reorganizes different ER membrane factors (BAP31, DnaJB14) to create a cytosol entry site [14,15]. Importantly, during cytosol entry, a membrane-associated disaggregation machinery (Hsc70, Hsp105, and DnaJB14) extracts SV40 into the cytosol in a step coupled to the further disassembly of the viral particle (step ii) [16]. From the cytosol, the partially disassembled viral particle transports into the nucleus and releases its genome in this compartment. Thus, an enzymatic reaction (localized in the ER lumen) followed by a mechanical force (encoded by the cytosolic disaggregation complex) is the cue combination used to uncoat this nonenveloped virus.

Another example of host cue and viral uncoating interplay is observed in the nonenveloped *Adenoviridae* (AdV) family. The species C viruses HAdV-C2/5 are the best-studied viruses from this family. While this virus is responsible for mild respiratory infections, it can also cause life-threatening diseases in immunocompromised individuals. AdV contains a highly stable capsid that encases its viral DNA genome [17]. Infection typically begins when the viral fiber and penton base proteins interact with the Coxsackievirus adenovirus receptor (CAR) and αvβ3/αvβ5 integrin coreceptors. These receptor interactions disrupt the viral architecture due to mechanical strain imposed on the virus. The mechanical tension results when the viral core capsid is tethered to stationary integrins, while the fibers are simultaneously bound to CAR molecules that actively drift on the plasma membrane. This capsid destabilization causes detachment of the fibers and exposure of protein IV (Fig 1D, step i) [18]. The structurally-primed virion then undergoes clathrin-dependent endocytosis to reach the endosome, where a pH-dependent step enables viral escape into the cytosol (step ii) [19]. Upon cytosol entry, AdV uses motor-driven, microtubule-based transport to reach the nucleus and dock on the nuclear pore complex. Here, a second mechanical force generated by the kinesin motor disassembles the virus, allowing the viral genome to be released into the nucleus (step iii) [20]. Hence, for the highly stable AdV, initial receptor engagement (leading to mechanical disruption) followed by a chemical cue and then a mechanical cue coordinately uncoat this virus.

Although the four examples illustrated above clearly demonstrate a complex relationship between viruses and host cues used during uncoating, a general uncoating strategy leading to genome delivery can nonetheless be observed. For many viruses, receptor engagement at the plasma membrane (that imparts viral conformational changes) is the first cue that primes viral uncoating. Proteolytic processing by host proteases localized on the plasma membrane (that also leads to viral structural alterations) can likewise be used to initiate uncoating before entry, as seen in the case of rotavirus and SARS-coronavirus (see Table 1 for more examples). After gaining entry into the host, low pH is often used as the subsequent cue to further uncoat the virus. However, enzyme- and/or chaperone-mediated cues can similarly be utilized within the host to trigger viral disassembly. Finally, in many instances, mechanical cues generated by molecular machines that convert the energy stored in nucleotides to mechanical forces, including motor proteins, disaggregation machinery, and the proteasome complex, are recruited to complete the uncoating process. It is interesting to note that, for the more stable AdV [21], mechanical cues that can impart powerful destabilizing forces disassemble these viral particles to cause genome release. In fact, the stability of viruses has also been implicated in the selection of host cues. For instance, the human nonenveloped RNA rhinovirus (HRV), a *Picornaviridae* family member, is classified into a major and a minor group based on receptor usage [22]. Because the major group (HRV-14/3) is thought to be more stable than the minor group (HRV-2/16), the major group requires uncoating by receptor-induced priming followed by low pH-mediated disassembly, while the minor group only requires chemical stimuli to uncoat (Table 1) [23].

While there are (and will continue to be) exceptions to the viral uncoating strategy that we have described in this short article, our intention is to organize the known disassembly mechanisms of approximately 30 different viruses from many virus families that are used to deliver the viral genome into the host. By depicting a general pattern, we hope this information may be useful for the broader virology community in deciphering the uncoating mechanism for a virus within the same family for which the uncoating strategy is known (see Table 1 for uncoating step marked as not determined [ND]). For instance, does the Merkel cell polyomavirus—the causative agent for the aggressive skin cancer Merkel cell carcinoma—exploit the same uncoating mechanism as other members of the *Polyomaviridae* family? Additionally, can we apply the uncoating program used by members of the *Coronaviridae* family to MERS coronavirus, a recently discovered member of this family that causes severe respiratory diseases? Finally, from a practical viewpoint, clarifying detailed viral uncoating mechanisms will continue to pave the way for identifying new therapeutic agents, as already successfully found in the discovery of many antiviral compounds that act primarily by inhibiting the viral uncoating process [24].
