# Correction: Viruses Utilize Cellular Cues in Distinct Combination to Undergo Systematic Priming and Uncoating

**DOI:** 10.1371/journal.ppat.1005712

**Published:** 2016-06-09

**Authors:** Madhu Sudhan Ravindran, Billy Tsai

There is a typographical error in page 4, line 21. The reference of "protein IV" should be changed to "protein VI". The sentence should read: This capsid destabilization causes detachment of the fibers and exposure of protein VI (Fig 1D, step i) [18].
